# Measuring patient satisfaction with four items: validity of the client satisfaction questionnaire 4 in an outpatient population

**DOI:** 10.1186/s12888-023-05310-w

**Published:** 2023-11-07

**Authors:** Henrik Pedersen, Tatiana Skliarova, C. Clifford Attkisson, Mariela L. Lara-Cabrera, Audun Havnen

**Affiliations:** 1 Division of Psychiatry, Nidaros Community Mental Health Centre, St. Olavs University Hospital, Trondheim, Norway; 2 Department of Mental Health, Faculty of Medicine and Health Sciences, Norwegian University of Science and Technology (NTNU), Trondheim, Norway; 3 Department of Psychiatry and Graduate Division, University of California, San Francisco, USA; 4 Tamalpais Matrix Systems, LLC, Chicago, USA; 5 Division of Psychiatry, Nidelv Community Mental Health Centre, St. Olavs University Hospital, Trondheim, Norway; 6 Department of Psychology, Norwegian University of Science and Technology (NTNU), Trondheim, Norway

**Keywords:** Patient satisfaction, Mental health, Client satisfaction questionnaire, Validity, Patient-reported experience, Quality of mental healthcare, Quality of care, Satisfaction with treatment

## Abstract

**Introduction:**

Patient satisfaction with mental health services has for several decades been considered an important component when evaluating service quality. It is often assessed in the context of monitoring quality of care, developing or evaluating newly implemented interventions or changes in practice. Because of this, patient satisfaction questionnaires are often added to longer questionnaire batteries, and shorter questionnaires are preferred to prevent respondent fatigue and non-compliance and to secure easy implementation. However, most studies use unvalidated patient satisfaction measures, making comparisons between studies difficult. Validation studies of short patient satisfaction measures are therefore warranted.

**Methods:**

The primary aim was to examine the construct validity and internal reliability of the Client Satisfaction Questionnaire-4 (CSQ-4) in a Norwegian outpatient mental health setting. A total of 467 patients were recruited from an outpatient psychiatric care clinic in Central Norway. The secondary aim was to examine an earlier proposed cutoff for classifying dissatisfied patients in this new population. A principal component analysis was conducted to evaluate factor structure, correlation analyses were conducted to test for predicted relationships, and Cronbach’s alpha was calculated to examine internal reliability.

**Results:**

The CSQ-4 showed a clear unidimensional structure with one factor explaining 80% of its variance. Its internal reliability was very high, with a Cronbach’s alpha of 0.92. As hypothesised this study found no statistically significant sex differences in satisfaction and no statistically significant association between age and satisfaction. Positive changes in symptoms during treatment and higher post-treatment functional impairment were associated with higher patient-reported treatment satisfaction scores, which indicates good construct validity.

**Conclusion:**

This is the first study to evaluate the CSQ-4 in a psychiatric population. The CSQ-4 demonstrated good structural validity and internal reliability and was correlated with outcome variables in terms of symptom change and post-treatment functioning. In sum, this indicates that the CSQ-4 is a good short alternative for evaluating patient satisfaction in routine outpatient mental health care.

## Background

For several decades, measuring patient satisfaction with mental health services has been considered an important aspect when evaluating service quality [1, 2]. It is a central component of patients’ experiences of treatment [1], and crucial when striving for patient-centered care [3]. This often leads patient satisfaction to being assessed in the context of monitoring quality of outpatient mental health care, evaluate interventions, or practice changes. Therefore, patient satisfaction measures are frequently added to already extensive questionnaire batteries, which have the risk of affecting compliance [4, 5]. In busy clinical settings, shorter questionnaires are preferred for efficiency, and the convenience of patients. These qualities, however, have to be balanced against the potential loss of psychometric properties compared to their longer counterparts [6].

Various systematic reviews on patient satisfaction questionnaires have emphasized the lack of well-validated measures, as well as a tendency of investigators to use short, pragmatic ad hoc measures, or measures used very infrequently [7, 8]. These research practices make comparison difficult. Indeed, without a meaningful comparison base, the scores have little to no practical utility [9].

The Client Satisfaction Questionnaire 8 item version (CSQ-8) [10], was initially developed for measuring satisfaction with services in a mental health outpatient setting [1] and is now one of the most used patient satisfaction questionnaires across many medical, psychiatric, and human service contexts [7, 8]. The CSQ-8 is a unidimensional measure of global satisfaction with high internal reliability, with a Cronbach’s alpha of 0.92-0.93 in its normative English sample [9]. Moreover, high scores on the CSQ-8 has been shown to correlate with treatment outcome, and adherence [9, 11, 12], while at the same time showing few correlations with demographic variables in routine care [1, 9, 12].

The CSQ-8 exists in over 50 languages [13], and has at this time been validated in two regional languages in the Philippines [14], French [15, 16], Thai [17], Dutch [18, 19], German [20], Japanese [21], Spanish [22] Castilian Spanish [23], and Norwegian [24]. Examinations of dimensionality and internal reliability of these translated versions have to a high degree resembled results found in the English norm sample [1, 9, 14, 15, 18, 19, 23–25].

Shorter versions of the CSQ-8 have been developed and are being used in clinical settings [26, 27]. For example, Greenfield [27], used four items from the CSQ-8 to assess satisfaction with university counselling services. These services offered students vocational counselling, helping them dealing with personal problems, or both. This four-item version (CSQ-4) showed internal reliability comparable to the CSQ-8 in similar populations [27]. Its association with other constructs, such as psychological distress, was comparable as well. In sum, the findings suggested that the CSQ-4 may be a valid shorter alternative to the CSQ-8.

However, the psychometric properties of the CSQ-4 have yet to be investigated outside of this context. Furthermore, the CSQ-4 has never been validated in a Norwegian population. Its use can therefore potentially introduce unwanted bias when treated as a shorter version of the Norwegian CSQ-8. Additionally, if a shorter version of the CSQ-8 is viable, this could make the assessment of patient satisfaction less burdensome and easier to implement in larger questionnaire batteries. Validated questionnaires are also essential to ensure that satisfaction scores have practical utility, and to enable comparisons between studies [9]. A validation study within a Norwegian mental health outpatient context is therefore warranted.

The primary aims of this study were to investigate the construct validity and internal reliability of the four-item measure CSQ-4 in a Norwegian mental health population. We expected the CSQ-4 to display similar psychometric properties as the CSQ-8 in similar contexts. More specifically, our predefined hypotheses were that the CSQ-4 would (1) show good structural validity shown by a strong one-factor structure, and (2) have high internal reliability indicated by a Cronbach’s alpha > 0.85. To further test construct validity, it was hypothesized that satisfaction of routine treatment would not be empirically associated with patient variables such as age and sex [24]. We, therefore, expected (3) no statistically significant difference in satisfaction between men and women, and no statistically significant relationship between age and satisfaction. We also expected satisfaction to be correlated with outcome variables such as functional impairment and change in psychiatric symptoms during treatment [9, 24]. Hence, we hypothesized that (4) the service satisfaction results would show a small to moderate positive correlation (stronger than *r* = .3) with treatment outcome, measured by symptom change during treatment and post-treatment functioning. The secondary aim was to examine Greenfield’s proposed cutoff in this new population.

## Methods

To answer the presented research questions, and to make the recent results as comparable as possible to earlier results, research context and data-collecting procedures mirror those outlined in Pedersen et al. [24]. A more concise description of this process is presented below. The study design and how the results are reported were guided by Consensus-based Standards for the Selection of Health Measurement Instruments-COSMIN [28].

### Participants and data collection

Data were collected from patients getting routine treatment at the outpatient clinics at Nidaros community mental health center, associated with the St. Olavs University Hospital, in Trondheim Norway. All patients received digital questionnaires a few days before their first session (T1) and after treatment termination (T2). Patient satisfaction was only measured at T2. The data collection period lasted from October 2021 to mid-February 2023.

Patients referred to the clinic were invited to participate through text message a few days before starting treatment. After following a hyperlink, they were presented with information about the project and a consent form. Upon receiving consent from patient participants, the questionnaires were answered. All answers and personal data were kept on a secure server, provided by the company Checkware AS.

To be included, patients had to provide informed consent and answer post-treatment questionnaires within the data collection period. All patients at the clinic were 18 years or older. Patients were excluded from data analysis if data regarding symptom severity or everyday functioning, either before or after treatment, or patient satisfaction after treatment was missing.

The final sample for this study consisted of 467 patients. The mean age was 31.33 years, and 67.4% identified as female, which mirrors the sample found by Pedersen et al., where 66% identified as female and the mean age was 29.9 [24].

The Regional Committee for Medical Research and Ethics in Norway (REK 2019/31,836) and the Norwegian Centre for Research Data (2019/605,327) formally approved this study. All participants were informed through the consent form that withdrawal was possible at any time without resulting in consequences.

### Measures

#### The client satisfaction Questionnaire-4

The CSQ-4 [27] is a shorter version of the CSQ-8 [9, 10] and consists of items 3, 6, 7, and 8 from the CSQ-8. The questionnaire consists of four items: “*To what extent has our program met your needs?”;* “*Have the services you received helped you to deal more effectively with your problems?*“; “*In an overall, general sense, how satisfied are you with the service you have received*”; “*If you were to seek help again, would you come back to our program?*^1^”. All items are measured on a four-point verbal anchor without a neutral position, ranging from “quite dissatisfied”, to “very satisfied”. Each item is scored from 1 to 4 leading to a score range of 4 to 16, where a higher score indicates higher satisfaction. Permission to use the CSQ-4, and quote its items, were granted before conducting this study, and the subsequent submission of this paper.

#### The patient health questionnaire 4

Symptom severity was measured by the Patient Health Questionnaire 4 **(**PHQ-4) [29, 30]. To measure the change in symptom severity throughout treatment, T1 scores were subtracted from T2 scores. The PHQ-4 is a four-item questionnaire measuring general psychiatric symptom severity with two questions derived from The Patient Health Questionnaire 9 [31] and The Generalized Anxiety Disorder Scale 7 [32] respectively. All four statements are preceded by “Over the last two weeks, how often have you been bothered by the following problems?”. Possible answers are presented on a four-point scale ranging from “not at all” to “nearly every day”. Each answer is coded from 0 to 3, leading to a score range of 0–12 [29]. A higher score indicates higher symptom severity. The PHQ-4 have been validated in a Norwegian population and demonstrated a two-factor structure [30].

#### The work and social adjustment scale

The Work and Social Adjustment Scale (WSAS) [33] measures how much a “problem” affects a person’s ability to function across five domains (work, home management, close relationships, and social- and private leisure activities). The questionnaire consists of five items, one for each domain (e.g., “Because of my [problem] my ability to work is impaired ‘0’ means ‘not at all impaired’ and ‘8’ means very severely impaired to the point I can’t work”), scored on a nine-point scale from 0 (not at all) to 8 (very severely), giving a score range of 0 through 40. A higher score indicates higher impairment and lower overall functioning. No time frame is used as a reference when answering. WSAS scores from T2 were used as a measure of post-treatment functioning.

### Statistical analyses

For the total score and individual items of the CSQ-4, mean (*M*) and standard deviation (*SD*) were calculated. To examine the score distribution pattern, the score distribution was presented visually. By calculating the percentage of minimum or maximum values, floor and ceiling effects were investigated guided by the suggested cutoff provided by Terwee et al., [34] of 15% to indicate problems.

Non-parametric tests were used due to negative skew and non-normality in satisfaction scores. To explore potential relationships between satisfaction and patient variables, such as age and sex, and outcome variables, such as changes in symptoms during treatment and post-treatment functioning, a two-tailed Mann-Whitney U-test and Spearman rank-order correlations were used. To enhance readability, scores were coded such that the hypothesised relationships between satisfaction and positive outcomes yielded a positive coefficient.

Cronbach’s *α* was calculated to examine internal reliability. A principal component analysis was conducted to investigate structural validity, after checking assumptions by using Bartlett’s test of sphericity and calculating the Kaiser-Meyer-Olkin measure of sampling adequacy. For factor analysis, the COSMIN guidelines [28] recommend a sample size of 7 times the number of items, and a minimum of 100 is recommended.

Due to the low number of items, respondents were removed if any missing occurred when calculating the total score of the CSQ-4, and the change in PHQ-4 scores. This led to 19 participants having incomplete CSQ-4 scores, and 18 participants having incomplete PHQ-4 scores. Six respondents did not answer any items on the WSAS. Two respondents had one missing response on the WSAS each, which was counted as a zero when calculating the total score. Missing data were less than the recommended cutoff of 5% on all variables [35], therefore, no imputations were done. In all analyses, missing values were excluded pairwise. Version 27 of IBM SPSS Statistics was used in all analyses.

## Results

### Descriptive statistics of satisfaction scores

**Fig. 1:**
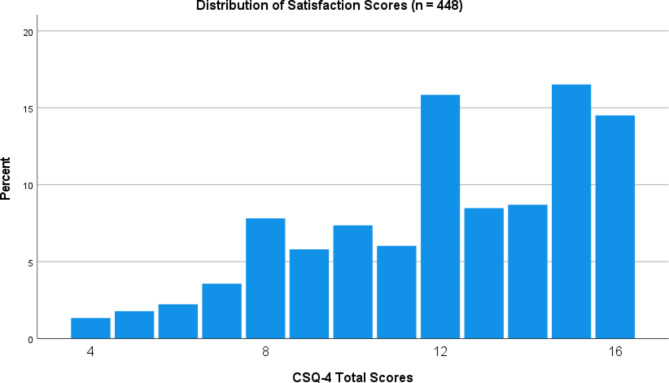
Distribution of satisfaction scores

67.4% of participants were female, and the average age was 31.33 years (*SD* = 10.22). Figure 1 shows the distribution of CSQ-4 total scores (*M* = 12.09, *SD* = 3.13, Median = 12). The scores were negatively skewed and not normally distributed, evaluated by inspection of Fig. 1 and a significant Shapiro-Wilk test (*p* < .001). Of the 448 patients in the sample without any missing items on the CSQ-4, 65 (14.5%) had a maximum score. Frequencies of different scores for individual items are presented in Table 1, while means and standard deviations for each item are presented in Table 2.

**Table 1:** Frequency of scores for each item in the CSQ-4 (N = 467)

	Frequency distribution of answers (%)					
items	1	2	3	4	Unanswered items (%)	
Item 3: Met needs	46 (9.9)	114 (24.4)	199 (42.6)	102 (21.8)	6 (1.3)	
Item 6: Improvement in self-efficacy	17 (3.6)	91 (19.5)	175 (37.5)	171 (36.6)	13 (2.8)	
Item 7: Overall satisfaction	29 (6.2)	95 (20.3)	158 (33.8)	173 (37.0)	12 (2.6)	
Item 8: Come back	16 (3.4)	74 (15.8)	185 (39.6)	176 (37.7)	16 (3.4)	

Note: CSQ-4 = Client Satisfaction Questionnaire 4; items are numbered as they appear in the CSQ-8.

**Table 2:** Individual items’ means, factor loadings, and internal reliability of the Norwegian CSQ-4

			Cronbach’s alpha = 0.92	
Items	Factor 1 loadings	*M* (*SD*)	Corrected Item-Total Correlation	Cronbach’s Alpha if Item Deleted
3. Met needs	0.885	2.78 (0.906)	0.79	0.90
6. Improvement in self-efficacy	0.911	3.11 (0.847)	0.84	0.88
7. Overall satisfaction	0.945	3.05 (0.922)	0.90	0.86
8. Come back	0.837	3.16 (0.821)	0.72	0.92
Total score		12.10 (3.13)		

Note: CSQ-4 = Client Satisfaction Questionnaire 4-item version; items are numbered as they appear in the CSQ-8; *M* = Mean; *SD* = Standard deviation

### Factor structure and internal reliability

The sample was deemed fit for a factor analysis as Bartlett’s Test of Sphericity was significant at the *p* < .001 level and the Kaiser-Meyer-Olkin Measure was 0.833. Factors were extracted by Kaiser’s criterion. Only one factor had an Eigenvalue exceeding 1 (Eigenvalue = 3.20), and all factor loadings exceeded 0.80. An overview of factor loadings is presented in Table 2. The extracted factor explained 80.1% of the variance.

The CSQ-4 showed very high internal reliability with a Cronbach’s alpha of 0.92. Higher scores could not be achieved if any of the items were to be deleted, and all corrected Item-total correlations were higher than the recommended cutoff of 0.70 [28]. All corrected item-total correlations and potential alpha values are presented in Table 2.

Alpha values from the PHQ-4 and WSAS were also calculated. For the PHQ-4, alpha values calculated in this study were 0.77 for the depression subscale, 0.82 for the anxiety subscale, and 0.78 for the entire scale. Cronbach’s alpha for the WSAS in this study was 0.82.

### Correlations and differences between groups

Aggregated information about age and sex is presented in Table 3. Spearman’s rank order correlation was computed to assess the relationship between age and satisfaction and between treatment outcome variables and satisfaction. No statistically significant relationship was found between age and satisfaction, *r* (446) = 0.007 [-0.089, 0.102], *p* = .889.

**Table 3:** Demographic information, symptom change, age, post-treatment functioning, and its correlations with satisfaction scores

		CSQ-4 total score			
		*n*	%	*r* (Mdn) [95%, CI]	*p*
Age	Mean (*SD*)	31.33(10.22)		0.007 [-0.089, 0.102]	0.889
Sex		467			
	Female	315	67.4	(12.50)	
	Male	152	32.6	(12.00)	
Symptom change (PHQ-4)		433		0.457 [0.530, 0.377]	< 0.001**
Post-treatment functioning (WSAS)		444		0.529 [0.594, 0.456]	< 0.001**

Note: CI = Confidence interval; CSQ-4 = Client Satisfaction Questionnaire-4; Mdn = Median; PHQ-4 = Patient Health Questionnaire-4; *SD* = standard deviation; WSAS = The Work and Social Adjustment Scale; Symptom change was calculated by subtracting T1 PHQ-4 scores from T2 PHQ-4 scores

A moderate positive correlation was found between CSQ-4 scores and a positive change in symptom severity (PHQ-4 scores), *r* (431) = 0.457 [0.530, 0.377], *p* < .001. A moderate to large positive correlation was found between the CSQ-4 and post treatment functioning (WSAS scores) at T2, *r* (442) = 0.529 [0.594, 0.456], *p* < .001. No statistical difference in satisfaction scores between men and women was found by the Mann-Whitney U test (*Mdn* = 12) and women (*Mdn* = 12.5), $\text{U}\left({\text{N}}_{\text{men}}\text{= 146, }{\text{N}}_{\text{women}}\text{ = 302}\right)\text{ = 21183.5,}\text{ z}\text{ = -0.676, }\text{p}\text{ = 0.499}$.

## Discussion

This is the first study to evaluate the CSQ-4 in a mental health population, and the first to explore its structural validity. We found the CSQ-4 to have good structural validity, internal reliability, and correlations to patient variables and outcome variables that are highly comparable to the CSQ-8 in the same context.

Compared to an earlier study, sample characteristics were almost identical with 67.4% female participants compared to 66.3%, and a mean age of 31.33 years compared to 29.97 years, which is also representative of the Norwegian psychiatric outpatient population as a whole [36]. Our mean and standard deviation on CSQ-4 total scores in this study are also approximately half of what was found in this earlier study on the CSQ-8, in the same psychiatric outpatient context.

The CSQ-4 showed a clear unidimensional structure, with one factor explaining most of the variance. We were not able to find any studies examining the factor structure of the CSQ-4, but several studies have examined the factor structure of its longer counterpart, the CSQ-8, all finding one factor [1, 9, 14, 15, 18, 19, 23–25]. Compared to the Norwegian CSQ-8, the extracted factor in this study explained marginally more of the questionnaire’s variance (80% compared to 74%) [24]. Because the CSQ-4 has four items, and we expect a one-factor structure, we deem our sample of 467 as more than adequate.

The internal reliability of the CSQ-4 was very high, with an alpha = 0.92. This is higher than found by Greenfield (CSQ-4 alpha = 0.86 to.88; CSQ-8 alpha = 0.88) [27]. However, the internal reliability of the CSQ-8 has been found to be higher when tested in a psychiatric population (0.92) [9]. The Norwegian CSQ-8 has shown even higher internal reliability in a psychiatric context (0.95) [24]. The lower value of the CSQ-4 in this study indicates that some redundant features may have been removed. Some redundancy may remain, however, as the alpha value is unchanged if item 8 is removed (unrounded values equal 0.917, and 0.921 if item 8 is removed).

No meaningful relationships were found between satisfaction and the examined patient variables. This is in line with previous research on Norwegian psychiatric outpatients, which found no sex differences in satisfaction and only a small to marginal statistical relationship between age and satisfaction [24]. It is worth noting, however, that there are contexts where sex or age differences may be more prevalent, than in routine care. Older people may feel alienated when receiving digital care, or care where interaction with unfamiliar digital devices is necessary [37]. Similarly, sex differences in satisfaction may occur in treatment programs developed for diagnoses where one sex traditionally has been underrepresented, where this underrepresentation may have influenced treatment programs to be more tailored to one sex [38]. If such differences are found by future research or in clinical practice, our results indicate that this might be due to an actual difference and not an artifact of the CSQ-4. However, this would warrant further investigation.

As hypothesized, we found moderate correlations between the CSQ-4 and outcome variables. Although somewhat higher, they are comparable to the associations between satisfaction measured by the Norwegian CSQ-8 and change in PHQ-4 scores (*r* = .355) previously reported [24]. This may indicate that satisfaction measures by the CSQ-4 is more related to positive treatment outcome, than the CSQ-8.

The scores were negatively skewed, with 14.5% achieving max scores, which is right on the threshold of 15%, which Terwee et al., have suggested as a cutoff indicator of ceiling effects [34]. We also found, however, that 15, the next highest total score, was the most common. Negatively skewed scores have long been a problem in patient satisfaction research [1], where it is hard to avoid sampling bias, because dissatisfied patients may be more likely to drop out, and less likely to complete questionnaires. Potential sampling bias is discussed below. Our findings nevertheless imply that the CSQ-4 does not suffer from ceiling effects, although more research on potential ceiling effects in the CSQ-4 is necessary.

### Implications and further research

Our results suggest that the construct validity of the Norwegian CSQ-4 is comparable to the Norwegian CSQ-8. As this shorter version is easier to implement in routine services than longer questionnaire batteries, this may make patient satisfaction assessment less demanding. The feedback from the CSQ-4 has the potential to help therapists towards a more patient-centred approach to mental health treatment and may provide useful insights from the patient’s perspective when developing and evaluating newly implemented interventions or evaluating changes in practice.

Some practitioners may be interested in identifying dissatisfied patients for additional feedback, either in research or in clinical practice. For this purpose, the pragmatic cutoff originally suggested by Greenfield [27], of at least two questions scored two or lower for dissatisfaction, seems reasonable. However, more research on this cut-off value is needed. There is also the possibility is to adding optional open questions answered in free text, where patients can write suggestions for improvement [1]. Earlier research on the CSQ-8 found that as many as one-third may answer such questions in addition to the questionnaire itself [24].

In light of our findings, we have several suggestions for further research. The literature is sparse when it comes to studies that have measured patient satisfaction at different time points. Such designs are necessary, however, to establish measurement error in terms of test-retest reliability, establish its responsiveness, and detect its smallest meaningful difference. To further examine its validity, cognitive interviewing may be used to investigate on what basis patients choose their answers in this context. Do they mainly, for example, have the interactions with their therapist in mind, or the services as a whole, like time spent on waiting lists or how they experienced the facilities themselves?

### Strengths and limitations

Limitations of this study include sample characteristics, an uncertain degree of anonymity experienced by the patients, and potential selection bias. Our sample consisted of predominately young female participants recruited from a psychiatric outpatient context, and the extrapolation of our findings should therefore be done with caution to other populations and contexts.

The questionnaire batteries used in this study are implemented in routine care at the community mental health center. This means that the degree of perceived anonymity is uncertain. However, this may not be a problem, as perceived anonymity does not seem to have a big impact on CSQ-8 scores in substance abusers, which may be transferable to the CSQ-4 in this context [39]. Furthermore, it is hard to estimate the degree of sampling bias in our study. Dissatisfied patients may, for example, be more inclined to drop out of treatment. This underscores a long-lasting challenge in patient satisfaction research [1] and emphasizes the importance of research measuring satisfaction at other points in time beyond treatment termination, which may be particularly vulnerable to bias.

We believe this study to also possess several strengths. Its design with regard to data collection, population, and context is identical to an earlier study evaluating the Norwegian CSQ-8 [24], which ensures comparability. It has a large and appropriate sample compared to the target population of mental health care outpatients. It is also the first study to test the factor validity of the CSQ-4, and the first to evaluate the CSQ-4 in a mental health context.

## Conclusions

In sum, the CSQ-4 shows highly comparable factor structure and internal reliability to the CSQ-8 in a Norwegian mental health population. Relationships between satisfaction and demographic variables, and between satisfaction and outcome measures were also comparable, which indicate that the CSQ-4 behave in the same way as the CSQ-8. Our results indicates that the CSQ-4 is a good shorter alternative to the CSQ-8. The CSQ-4 has the potential to lessen the burden of measuring patient satisfaction for clinical and research purposes, which may increase the frequency of patient satisfaction assessments in the future. More research is needed, however, measuring patient satisfaction over multiple time points to assess test-retest reliability, responsiveness, and meaningful change.

## Data Availability

The dataset analysed during the current study is not publicly available, but it can be available from the corresponding author on reasonable written request.

## Abbreviations

CSQ: The Client Satisfaction Questionnaire
CSQ-4: The Client Satisfaction Questionnaire 4-item version
CSQ-8: The Client Satisfaction Questionnaire 8-item version
*M*: Mean
Mdn: Median
PHQ-4: Patient Health Questionnaire 4-item version
*SD*: Standard Deviation
REK: Regionale komiteer for medisinsk og helsefaglig forskningsetikk. *Translated*:Regional Ethical Committee
WSAS: The Work and Social Adjustment Scale

## Footnotes

1 The CSQ-4 is copyright intellectual property, and its use is limited to individuals and agencies who have received express permission from the copyright holder to use the scale and who meet the requirements pertaining to use. Information about permission to use the instrument can be found at csqscales.com.
